# Supplementary material for: Host-pathogen interaction: Enterobacter cloacae exerts different adhesion and invasion capacities against different host cell types
Source: PLoS One. 2023 Oct 24;18(10):e0289334. doi: 10.1371/journal.pone.0289334 (PMC10597508; doi:10.1371/journal.pone.0289334)
Supplement: S1 Data — (DOCX) [file pone.0289334.s001.docx]

**SUPPLEMENTARY DATA**

**Host-pathogen interaction: *Enterobacter cloacae* exerts different adhesion and invasion capacities against different host cell types**

Elisabet Frutos-Grilo^1, #a^, Vanessa Kreling^2^, Andreas Hensel^2*^ and Susana Campoy^1^

^1^ Departament de Genètica i de Microbiologia, Universitat Autònoma de Barcelona, Bellaterra, Spain.

^2^ Institute of Pharmaceutical Biology and Phytochemistry, University of Münster, Münster, Germany.

^#a^ Current Address: Centre for Genomic Regulation (CRG), The Barcelona Institute of Science and Technology, Dr. Aiguader 88, Barcelona 08003, Spain.

* Corresponding author

E-mail: [ahensel@uni-muenster.de](mailto:ahensel@uni-muenster.de) (AH)

ORCID ID: 0000-0001-7734-7983

**S1. Figure** **Bacterial load present of *E. cloacae* growing in DMEM media with 10% FCS at different times (0, 1 h, and 2 h) in absence or presence of 100 µg/mL gentamicin.** Values represent the mean ± SD of three independent assays. P < 0.01 was determined as statistically high significant (**) compared with the first column.

**S2. Figure** **Influence of filtered bacterial culture medium from *E. cloacae* at different OD on the cell viability (MTT assay) of different eukaryotic cells.** Data indicate relative viability [%] of (A) T24, (B) Caco-2, (C) A-431, (D) A-498, and (E) A549 cell lines within the specified conditions (OD inoculum at 0.2, 1, and 4; Incubation time 0, 30 min, 1 h, 3 h, and 5 h) related to the untreated control. UC (untreated control): cells cultivated in DMEM, supplemented with FCS. Cells cultivated in contact with 90% DMEM + 10% DMSO and 50% DMEM + 50% PBS are represented as “DMSO” and “DC”, respectively. Values represent the mean ± SD of three independent assays with 6 technical replicates in each assay. One-way ANOVA and Dunnett’s multiple comparison test (comparing all columns with the DC one) were used for statistical analysis. **: *p* < 0.01 (represented only in the columns with a lower median than the DC column in each case).

**
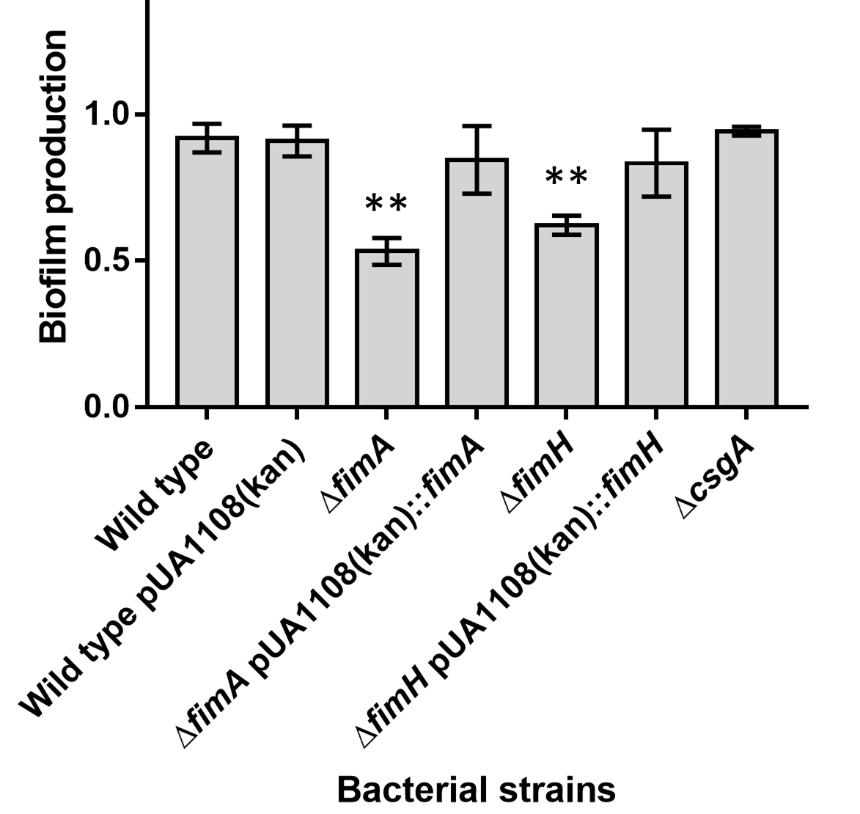
**

**S3. Figure Biofilm formation of wild type and fimA, fimH and csgA mutants.** Values represent the mean ± SD of three independent assays. P < 0.01 was determined as statistically high significant (**) compared to the wild type.

**
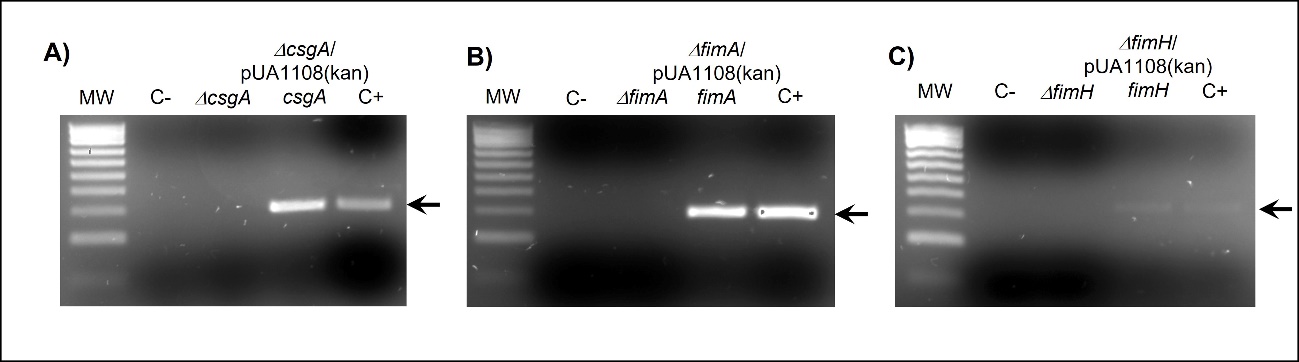
**

**S4. Figure Validation of the complementation of the Δ*fimA,* Δ*fimH,* and Δ*csgA* mutants with the corresponding wild-type gene.** Agarose gel electrophoresis of the RT-PCR of *Enterobacter* *csgA* (A), *fimA* (B), and *fimH* (C) mutants and the mutant strains containing the pUA1108 with the corresponding wild-type gene. In all cases, RNA was extracted from the mutant and complemented strains, and the cDNA was obtained using the NZY First-Strand cDNA Synthesis Kit (nyztech) and the suitable oligonucleotides. Each cDNA was afterward used for PCR amplification. As expected, no amplification is observed in the mutant strains, where the gene was deleted, but there is band when the corresponding plasmid is present. The expression of *csgA, fimA*, and *fimH* genes is confirmed by the presence of a 302 bp, 318 bp, and 349 bp amplification band, respectively. As positive controls (C+), for each gene, the PCR using *Enterobacter* wild-type genomic DNA was included. Also, as negative controls (C-), RT-PCRs with no cDNA were shown. MW, is the Ladder V molecular weight (nzytech).


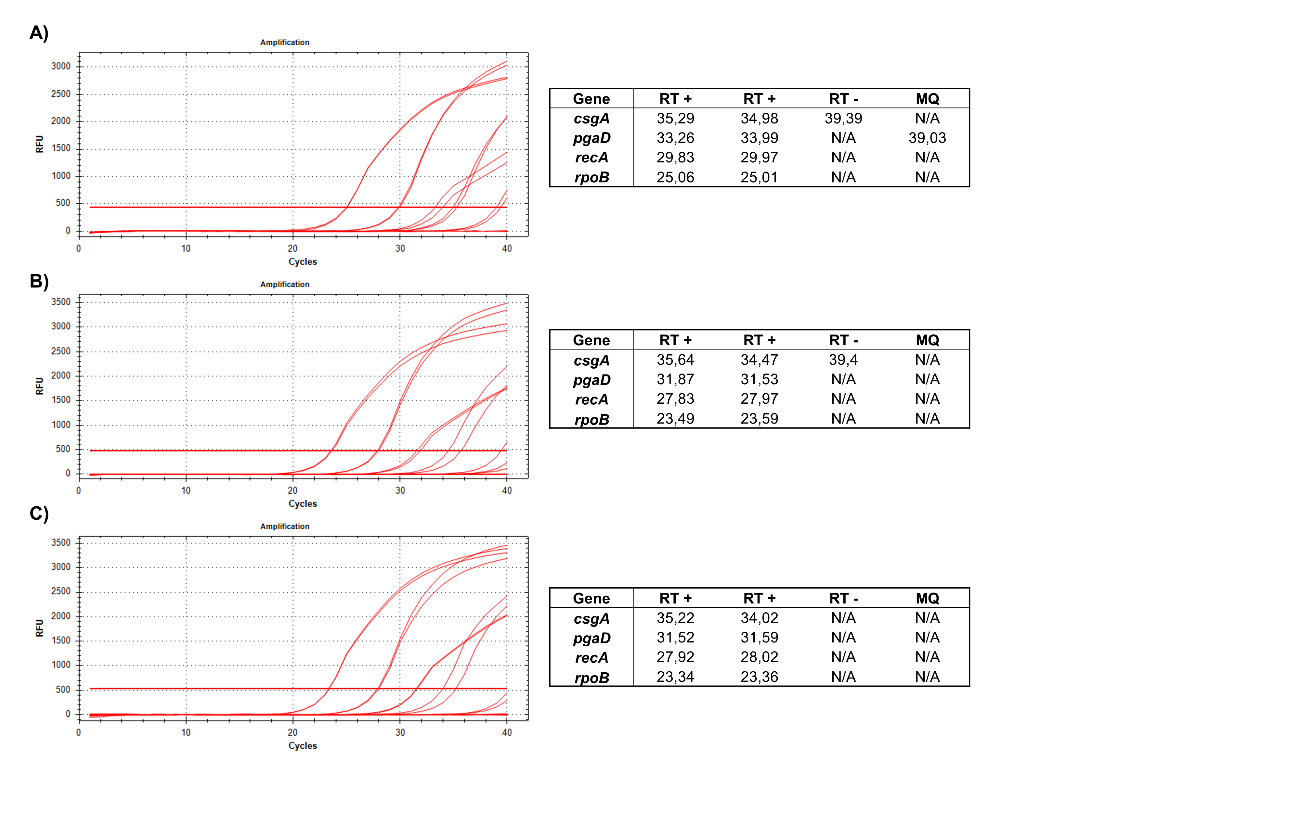


**S5 Fig. Validation of the expression of curli fimbriae and biofilm formation genes.** Real Time quantitative PCR (RTqPCR) of *csgA* gene, which form part of the curli appendage [1], and *pgaD* gene, which promotes cell-to-cell adhesion and surface attachment [2] of *E. cloacae* during adhesion assay conditions. In all cases, RNA was extracted from the mutant and complemented strains using RNeasy Kit (Qiagen) following manufacturer’s protocol. cDNA was obtained using the NZY First-Strand cDNA Synthesis Kit (NZYtech) and random hexamers and oligo(dT)_18_ primers. Each cDNA was afterward used for RTqPCR amplification (RT+). A, B and C represents each biological replicate, showing in the left side, the given amplification plots. The table from the left side represents the given Ct results analysed by Bio-Rad CFX Maestro software. As expected, no amplification (or mostly) is detected in those wells were non-transcribed cDNA (RT-) or MQ water (MQ) were introduced as template. Two technical replicates were used in these experiments. *recA* and *rpoB* genes were selected as housekeeping genes.

[1] Fronzes R, Remaut H, Waksman G. Architectures and biogenesis of non-flagellar protein appendages in Gram-negative bacteria. EMBO J. 2008;27(17):2271-80. doi: 10.1038/emboj.2008.155

[2] Ballén V, Cepas V, Ratia C, Gabasa Y, Soto SM. Clinical *Escherichia coli*: from biofilm formation to new antibiofilm strategies. Microorganisms. 2022;10(6):1103. doi: 10.3390/microorganisms10061103

## Material and methods. Influence of cell-free supernatant on host cell viability (MTT assay)

The assay was performed as described before [1]. To obtain cell-free supernatants (CFS), *E. cloacae* overnight grown LB-plate culture was incubated at 37 ºC and 10 % CO2 and was diluted in DMEM supplemented with 10 % FCS (Merck) (DMEM/FCS) at different conditions (0, 30 min, 1 h, 3 h, and 5 h of incubation periods, OD_640nm_ of 0.2, 1, and 4). Resulting cultures were centrifuged to discard the pellets and the supernatant was filtered by use of 0.22 µm pore size filters.

Subsequently, 5 ×10^4^ eukaryotic cells/well were seeded into 96-well plates and incubated at 37 ºC (CO_2_ content in the atmosphere according to the respective cell line), for 48 h. Cell monolayer at 90 to 100 % of confluency were washed 1 × with PBS and 100 µL of fresh DMEM/FCS and 100 µL of previously prepared *E. cloacae* supernatants were added. Negative control well (DMSO): 20 µL of DMSO diluted in 180 µL of DMEM/FCS; positive control well (UC): 200 µL of DMEM/FCS; diluted control well (DC): 100 µL DMEM/FCS and 100 µL of PBS 1×. Supernatants and eukaryotic cells were incubated during 48 h at 37 ºC/ 5-10 % CO_2._ Cells were washed with 200 µL/well of PBS. 50 μL of MTT reagent (3-[4,5-dimethylthiazole-2-yl]-2,5-diphenyltetrazolium bromide, 5 mg/mL in PBS, Sigma Aldrich) were added under light protection, followed by incubation at 37 °C and the corresponding CO_2_ level for 4 h. The enzymatic reduction of MTT) to an insoluble formazan derivative is catalyzed by mitochondrial succinate dehydrogenase. Hence, the MTT assay is dependent on mitochondrial respiration and indirectly serves to assess the cellular energy capacity of a cell [2]. The MTT reagent was removed and 50 μL of DMSO were added to dissolve the generated formazan crystals. After shaking for 10 min, the resulting colour was measured at λ = 595 nm against a reference wavelength of λ = 690 nm using Tecan Sunrise microplate reader. Three independent biological experiments with each 6 technical replicates were used for the posterior analyses.

[1] Mosmann T. Rapid colorimetric assay for cellular growth and survival: Application to proliferation and cytotoxicity assays. J Immunol Methods. 1983 Dec;65(1–2):55–63.

[2] Chacon E, Acosta D, Lemasters JJ. Primary cultures of cardiac myocytes as *in vitro* models for pharmacological and toxicological assessments. In: *in vitro* methods in pharmaceutical research. Elsevier; 1997. p. 209–23.

**S1 Table. Primers used in this work.**

**S1 File**. **Protein alignment/profile-HMM of *E. cloacae* ATCC 13047 coding sequence database.** All coding sequences of *E. cloacae* ATCC 13047 (NCBI ID: NC_014121.1) genome are shown. The PFAM ID is represented for each sequence in order to reveal which protein family it belongs to using the HMMsearch online server [1].

[1]. HMMER Website version 2.41.2. Biosequence analysis using profile hidden Markov Models. [cited 4 March 2022]. In: EMBL.EBI [Internet]. Available from: https://www.ebi.ac.uk/Tools/hmmer/.

| **Name** | **Sequence (5’🡪3’)** | **Usage** |
| --- | --- | --- |
| *fimA_GmF* | atgaaactcagcaacattgcttctactgttattgcttcactggccctggtcgcgggtgccgctcacgctgccgatccggttaTTAGGTGGCGGTACTTGG | Gentamicin cassette amplification flanked with beginning and ending homolog regions of *fimA* |
| *fimA_GmR* | ttattcgtatttcataacgaaagtggcgtcagcgtttgcctggcctggttcagtggtcgcagcagtggctttataacgcgcgGCGCGGAACCCCTATTTG |  |
| *fimH_GmF* | atgcgcagtttacgcaatgtgtttagcttagcgctgctctgcagcacgatgcccgcgtgggcgacggtttgccagaacgccaTTAGGTGGCGGTACTTGG | Gentamicin cassette amplification flanked with beginning and ending homolog regions of *fimH* |
| *fimH_GmR* | ttaatcgtaatccacgcgcagatagccgcgagaggtgaaccggccttctgctggcttattccccgtaatgctcaccggccagGCGCGGAACCCCTATTTG |  |
| *csgA_GmF* | atgaaatttatcaaagtggcagctcttgcagcaatcgtcgtttctggtagtgctatggccggtctcattgatcaaggtggctTTAGGTGGCGGTACTTGG | Gentamicin cassette amplification flanked with beginning and ending homolog regions of *csgA* |
| *csgA_GmR* | ttaatactgataagctgcagcgtggttaccaaaaccaatctgggtaacattgaccgtagaattagacgcagtctgatcaaccGCGCGGAACCCCTATTTG |  |
| *fimA_GmF* | GTCTCCTCCGAAATAAG | Amplification at ≈ 100bp upstream and downstream of *fimA* |
| *fimA_exF* | GTTAACAGCAGTAGTGTTC |  |
| *fimH_exF* | AGTCCCAGTGTCGTG | Amplification at ≈ 100bp upstream and downstream of *fimH* |
| *fimH_ExR* | TATTGATCTCGCCCAG |  |
| *csgA_ExF* | GCAAGGAGAATTAAGGTATTC | Amplification at ≈ 100bp upstream and downstream of *csgA* |
| *csgA_ExR* | AAACGCAGTTGTAGTG |  |
| *fimA_F* | acacaggaaacagtacatatgATGAAACTCAGCAACATTG | Amplification of *fimA* suitable for HiFi ligation |
| *fimA_R* | ccggggaattccggggatccTTATTCGTATTTCATAACGAAAG |  |
| *fimH_F* | acacaggaaacagtacatatgATGCGCAGTTTACG | Amplification of *fimH* suitable for HiFi ligation |
| *fimH_R* | ccggggaattccggggatccTTAATCGTAATCCACG |  |
| *csgA_F* | acacaggaaacagtacatatgATGAAATTTATCAAAGTGGCAG | Amplification of *csgA* suitable for HiFi ligation |
| *csgA_R* | ccggggaattccggggatccTTAATACTGATAAGCTGCAG |  |
