# Supplementary material for: Host-pathogen interaction: Enterobacter cloacae exerts different adhesion and invasion capacities against different host cell types
Source: PLoS One. 2023 Oct 24;18(10):e0289334. doi: 10.1371/journal.pone.0289334 (PMC10597508; doi:10.1371/journal.pone.0289334)
Supplement: Attachment — Submitted filename: Response to Reviewers Version 2 ELISA_SC 05062023.docx [file pone.0289334.s002.docx]

Response to Reviewers

Dear Editor,

Thank you for providing us with the opportunity to revise our manuscript based on the valuable feedback from the reviewer. We appreciate the time and effort that the reviewer has taken to carefully evaluate our work.

We have addressed all the concerns in the revised manuscript. Below this text, we provide a detailed response to each of the points raised, in bold. We hope that these revisions meet with the satisfaction of the reviewers and the editorial board. Once again, we thank the reviewers and the editor for their valuable feedback and guidance, which have helped to improve the quality of our work.

Sincerely,

**Journal requirements:**

1. Please ensure that your manuscript meets PLOS ONE's style requirements, including those for file naming. The PLOS ONE style templates can be found at https://journals.plos.org/plosone/s/file?id=wjVg/PLOSOne_formatting_sample_main_body.pdf and [https://journals.plos.org/plosone/s/file?id=ba62/PLOSOne_formatting_sample_title_authors_affiliations.pdf](about:blank)

We have changed the file naming and other issues in order to ensure the PLOS ONE’s style requirements.

2. Thank you for submitting the above manuscript to PLOS ONE. During our internal evaluation of the manuscript, we found significant text overlap between your submission and previous work in the [introduction, conclusion, etc.].

We would like to make you aware that copying extracts from previous publications, especially outside the methods section, word-for-word is unacceptable. In addition, the reproduction of text from published reports has implications for the copyright that may apply to the publications.

Please revise the manuscript to rephrase the duplicated text, cite your sources, and provide details as to how the current manuscript advances on previous work. Please note that further consideration is dependent on the submission of a manuscript that addresses these concerns about the overlap in text with published work.

[If the overlap is with the authors’ own works: Moreover, upon submission, authors must confirm that the manuscript, or any related manuscript, is not currently under consideration or accepted elsewhere. If related work has been submitted to PLOS ONE or elsewhere, authors must include a copy with the submitted article. Reviewers will be asked to comment on the overlap between related submissions ([http://journals.plos.org/plosone/s/submission-guidelines#loc-related-manuscripts)](about:blank).]

We will carefully review your manuscript upon resubmission and further consideration of the manuscript is dependent on the text overlap being addressed in full. Please ensure that your revision is thorough as failure to address the concerns to our satisfaction may result in your submission not being considered further.

All text has been carefully read to identify duplications using plagiarism checker servers (duplichecker.com and plagiarismdetector.net), trying to minimize the overlapping text with other works despite being referenced. We have realized an unreferenced and duplicated sentence, hence we have rephased it and referenced (LINE 70-72). However, we have not found any other sentence suspected to be duplicated. We would appreciate if you detect any other concerning sentence, let us know.

3. We note that you have included the phrase “data not shown” in your manuscript. Unfortunately, this does not meet our data sharing requirements. PLOS does not permit references to inaccessible data. We require that authors provide all relevant data within the paper, Supporting Information files, or in an acceptable, public repository. Please add a citation to support this phrase or upload the data that corresponds with these findings to a stable repository (such as Figshare or Dryad) and provide and URLs, DOIs, or accession numbers that may be used to access these data. Or, if the data are not a core part of the research being presented in your study, we ask that you remove the phrase that refers to these data.

Please review your reference list to ensure that it is complete and correct. If you have cited papers that have been retracted, please include the rationale for doing so in the manuscript text, or remove these references and replace them with relevant current references. Any changes to the reference list should be mentioned in the rebuttal letter that accompanies your revised manuscript. If you need to cite a retracted article, indicate the article’s retracted status in the References list and also include a citation and full reference for the retraction notice.

The included “Data not shown” has been eliminated from the manuscript but we described the results (LINES 211 AND 297) which reads now as follows: *Non-infected controls were prepared in a similar way, but without addition of bacteria to the cells; as expected, no FITC fluorescence was observed.*

**Reviewers’ comments:**

I. 245-259, Fig. 1 as well as l. 399-400: This reviewer does not think the wording is correct here. The disadvantage of the FACS-based method for quantifying bacterial adherence is that unfortunately no absolute numbers of adherent bacteria are obtained. This would have been more helpful here. This reviewer believes that the relative adherence determined with an MOI of 10:1 does fit the data obtained with an MOI of 100:1. Just because fewer bacteria were used does not mean they are better at binding. The relative adherence seems to be proportional to that obtained for the 100:1 MOI. The binding of *E. cloacae* to T24 and A-498 cells is thus significantly better than to Caco-2 and A-549 cells and about twice as good as to A-549 cells. The data would be even more convincing if the authors would, as a proof of principle, exemplarily quantify the number of adherent bacteria by CFU determination according to the quantification of the invasive bacteria.

This is a very interesting issue, thanks for this comment. The experiments indicated different sensitivity of the different cell lines. While T24, A-498 and A-549 cells were strongly infected by the bacteria at BCR 100:1 nearly no interaction has been shown for Caco-2 and A-431 cells (Fig. 1A). We have increased the BCR to 500:1 for Caco-2 and A-431 and again we do not find relevant fluorescence intensity (Fig. 1C). From these data we can clearly conclude that these cell lines are much less sensitive to E. cloacae compared to the bladder, kidney and lung cells. For the sensitive cell lines we used also a lower BCR of 10:1 but in this experimental set up we detect only very low intensity (Fig. 1B). This might have two reasons: Either a certain bacteria density if needed for infection or the fluorescence intensity is very low, as only 10% of the labelled bacteria load has been used in this experiment. We have inserted these both points into the text – our impression is that we are working in this protocol at the analytical limit of qunatitatioin in the FACS assay. Alternatively CFU counting could be performed, which normally works very well, but at this low BCR standard deviations are so high that a clear and statistical evaluation cannot be done. Concenring the advantages and disadvantages of the FACS assay raised by the referee, we have the feeling that flow cytometric data are getting pretty good results for adhesion assays, as this protocol has been established for quantitative evaluation of new adhesion and invasion blockers since some years, leading to less standard deviations as we normally see in CFU counting.

The respective section in the MS reads now as follows: “….Using this protocol, strong interaction of FITC-labelled *E. cloacae* with T-24 bladder cells, A-498 kidney cells, and A-549 lung cells was observed at BCR 100:1. A-431 and Caco-2 cells turned out to be not sensitive against the bacterial treatment under these conditions (Figure 1A). Additionally, it got obvious that lower titers of *E. cloacae* load (BCR of 10:1) were not enough to initiate relevant bacterial adhesion to T-24, A-498 and A-549 cells, which (Figure 1B). This could be because either a certain amount of bacteria is needed to infect the host cells or it is simply due to a very low fluorescence intensity as only a low amount of bacteria has been used in this protocol. Considering that *E. cloacae* displayed only low interaction with Caco-2 and A-431 cells at BCR 100:1, also BCR 500:1 was also employed for the adhesion assays with these cell lines (Figure 1C). However, this only results in a slight increase in fluorescence intensity, indicating that also increased bacteria level will not leed to more adhesion to Caco-2 and A-431 cells.”

This reviewer is not convinced that a minimal load of inoculum or quorum sensing is required here.

We are totally agree with the reviewer’s opinion and we have removed the sentence “*The differences in the obtained results for these three tissues using the BCR of 10:1 and 100:1 were not proportional, demonstrating that a minimal load of inoculum is required, where quorum sensing could be discussed for this phenomenon.”* in order to avoid to talk about non demonstrated facts.

II. 367-371, Fig. 6: The authors describe that subcloning of *fimA*, *fimH* and *csgA* in trans can restore the bacterial adherence phenotype to T-24 cells. The data presented looks nice, but it would be even nicer if the authors could provide additional evidence (SDS Page, ELISA, …) that indeed the subcloned gene results in expression of the corresponding gene product.

We provide RT-PCR assays showing the expression of the *fimA, fimH*, and *csgA* genes. As is shown in the new Suppl. File S4, in all mutant strains, and as expected, no RNA of the deleted gene (*csgA, fimA*, or *fimH*) is detected. However, the expression of these genes is only detected when the pUA1108 (kan) plasmid carrying the corresponding wild-type gene is incorporated into the mutant strain. These results, together with those in Figure 6 of the manuscript unequivocally indicate that the presence of the corresponding plasmid restores the expression of each deleted gene.

III. 423-430: The authors should phrase this sentence more carefully. According to the genome sequence, isolate ATCC 13047 has several fimbrial determinants. What do the authors know about the expression of different adhesins under the conditions that they have used? Can they prove that other fimbriae than type 1 fimbriae are not involved in bacterial adherence to T-24 or A-498 cells?

We don’t know about the expression of other adhesins present in the strain, for this reason we recommend further studies in order to investigate it. We rephrase the sentence: “As the fimbrial adhesins from UPEC are typical mannose-binding proteins, we hypothetise that the interaction of E. cloacae with T-24 bladder cells is due to a recognition of highly mannosylated uroplakin Ia and IIIa of the bladder cells, known to be the binding partner of the lectin-like domain of FimH from UPEC [54].”

Instead of:

“As the fimbrial adhesins from UPEC are typical mannose-binding proteins, we assume that the interaction of E. cloacae with T-24 bladder cells is due to a recognition of highly mannosylated uroplakin Ia and IIIa of the bladder cells, known to be the binding partner of the lectin-like domain of FimH from UPEC [54].”

IV. 432-434: Please phrase more carefully. Can the authors show that strain ATCC 13047 expresses curli under the test conditions?

In order to demonstrate that *E. cloacae* ATCC13047 expresses curli under the test conditions, we provide a RT-qPCR results in S5 Figure of the Suppl. Data.

The study would benefit from more careful visualization of adherent bacteria by electron microscopy or atomic force microscopy.

We concur with the reviewer's viewpoint and the study would benefit from more careful visualization of adherent bacteria by electron microscopy or atomic force microscopy. However, bacterial adhesion and invasion has been proven using confocal images, as we do, in different publications (1 doi.org/10.1016/S0076-6879(95)53016-9; 2 10.1038/s41598-020-63714-0; 3 10.1128/AEM.03323-12). Moreover, with the confocal images we have a 3D representation of the adhesion and invasion events (as presented in the Figures 2 and 5 of the present MS).

Another convincing proof that indeed adhesion mediated by type 1 fimbriae would be to demonstrate that bacterial adherence can be blocked by prior pre-incubation of the bacteria with e.g. methyl-α-D-mannopyranoside.

This is of course a good positive control, which definitely will be integrated into future investigations.

It would be interesting to see if the *fimA*, *fimH* and *csgA* mutants differ from the wild type strain in their ability to form biofilms.

We are agree with the reviewer interests and we add biofilm results of the ability of *fimA*, *fimH* and *csgA* mutants to form biofilm. The image has been added to the Supplementary Data S3) into the manuscript.
