# Supplementary material for: Host-pathogen interaction: Enterobacter cloacae exerts different adhesion and invasion capacities against different host cell types
Source: PLoS One. 2023 Oct 24;18(10):e0289334. doi: 10.1371/journal.pone.0289334 (PMC10597508; doi:10.1371/journal.pone.0289334)
Supplement: Attachment — Submitted filename: Referees comments and action of the authors.docx [file pone.0289334.s003.docx]

**Referees comments and action of the authors**

| Comment of reviewer | Action of authors |
| --- | --- |
| Reviewer #2: The article entitled ‘Host-pathogen interaction…..’ by Hensel et al. describes mechanism of pathogenicity of E. cloacae using different cell lines. Despite work on several pathogens, the pathogenicity of E. cloacae, a member of ESKAPE organisms, is still under-explored. **This work investigates the adhesion and invasion of E. cloacae in cell lines which is important for understanding the pathogenicity of nosocomial pathogens. The work is well designed and executed**. **The authors have also incorporated the suggestions of other reviewers**. However, I have few minor concerns which can be corrected or addressed. | ´  Thanks for the positive evaluation! |
| Abstract Line 35-36: Correct ‘extend’ to extent. Do the same across the text wherever it applies. | Has been changed as requested (also over the MS, 5 ×) |
| Line 126, 169: O.D of 4 or 0.4. Please confirm. 4 O.D seems too high.  Line 128: is it 8 or 0.8 OD? | The OD values of the cultures have been determined after appropriate dilution to enter a valid photometric range, but a given then for the undiluted text sample. |
| Line 269: How do you rule out the possibility of T6SS-mediated cytotoxicity as you have used CFS? E. cloacae is known to possess T6SS, which causes contact-dependent cytotoxicity. | Thanks for this advice. In this experiment we have shown that the bacterial adhesion of E. cloacae to the different host cells is not disturbed by soluble exotoxin secretion by using cell free and filtered culture supernatants of the bacteria at different ODs. This experiments did not show any influence on the different host cells, indicating that no soluble bacterial toxins disturb the assay. Unfortunately we wrote in the old version the phrase “… to show that the adhesion of E. cloacae to the host cells is not disturbed by contact associated cytotoxicitry or by exotoxin secretion”. The reviewer is absolutely right, T6SS would be a problem in the cell systems, but as we used in this experiment soluble cell free systems this secretion system is not part of the test solutions. We have changed the respective paragraph and deleted “by contact-associated cytotoxicity”. |
| Fig 3: What is on Y axis | Y axis described the relative adhesion of FITC-labeled bacteria at BCR 500:1 to different host cells at different days. The Relative data indicate the fluorescence related to uninfected cells, which is stated in the legend.  As mentioned in the corresponding Material and Methods “The relative adhesion was calculated from the measured median of the infected cells compared to that of the control without bacteria“, we have divided the given fluorescence of the infected cells, by that from the non infected control. So we have X times more fluorescence when we are adding the bacteria because it is adhered onto the epithelial cells.  We have added this to the legend of Fig. 3, which now reads as follows:  “**Fig 3.** **Relative adhesion of FITC-labeled *E. cloacae* at BCR 500:1 to Caco-2, HT29 and HT29-MTX at different days of post-seeding (2, 10, and 25).** Relative data indicate the fluorescence related to uninfected cells and has been calculated from the median of the infected cells compared to that of the control without bacteria. Values represent the mean ± SD from three independent assays and X-bar begins at Y = 1.” |
| Line 430: Italicize species name. | Has been changed as requested |
| **Reviewer #3:** I am seeing this manuscript for the first time (after it has already been revised, obviously). Overall, this is robust work and the previous referee comments have been addressed satisfactorily. I have some smaller comments to manuscript organisation and to phrasing/language: | Thanks for the positive comments! |
| 1) Manuscript Organisation: supplementary material. I find it porblematic to "hide" normal figures in the supplementary material in an online journal that has no restriction on the number of figures for the main text. I feel that in PLOS ONE and comparable journals, supplementary material should be restricted to large datasets, movies, etc that cannot be displayed in a PDF properly. All other data can only fall into two categories: (A) important - then show it in the main manuscript and reference/discuss it. Or (B) not important: then do not show it and do not talk about it in the manuscript. | This is a difficult question which should be decided by the editor or by the journals management. From our point of view, the Suppl Material provides access of data to the reader of the MS, which is not absolutely essential to know, but prove and validate experimental data which again validate the main experiments and results (e.g. pre-experiments, validation studies, dose finding). Or they simply get access to some very special features of the materials used (in our cases detailed description of the E. cloacae mutants) or similar data, which are essential for good science but only interesting for a reader f he really wants to go in the lab experiments or wants to repeat experiments in his own lab. From our point of view the Suppl. Data provide a nice help for interesting scientists in the daily lab routine.  Of course we easily can insert all our Suppl Data into the main MS, but we have the feeling that then the text could be overloaded and the main messages might be difficult to access.  If the editor wants us to do this, we are happy to revise, but according to our feeling and experience in publishing > 300 papers it could be clearer to have a separation into the main data set and a Suppl. Data File. However, we are very open to potential improvements. |
| 2) phrasing: I ma not very happy with the wording related to the binding/interaction data between cells and bacteria. It feels a bit "off" to me to say "A-431 and Caco-2 cells turned out to be not sensitive against the bacterial treatment" - what do you mean by "sensitive"? What you are observing is bacteria binding to cells, not cells 'reacting' to the bacteria, correct? I suggest to find a better way to describe the physical interaction (or non-interaction) that is observed here. This might apply to other sections of the text too. | Thanks for this advice, this is true.  We have changed the text accordingly, which reads now as follows:  “….Using this protocol, strong interaction of FITC-labelled *E. cloacae* with T-24 bladder cells, A-498 kidney cells, and A-549 lung cells was observed at BCR 100:1. A-431 and Caco-2 cells turned out to have no or only limited binding capacity against the bacterial treatment under these conditions and no adhesion or docking of the bacteria is observed (Fig 1A).” |
